# Nitric Oxide Is Associated With Heterosis of Salinity Tolerance in *Brassica napus* L.

**DOI:** 10.3389/fpls.2021.649888

**Published:** 2021-05-28

**Authors:** Yihua Zhang, Pengfei Cheng, Jun Wang, Dyaaaldin Abdalmegeed, Ying Li, Mangteng Wu, Chen Dai, Shubei Wan, Rongzhan Guan, Huiming Pu, Wenbiao Shen

**Affiliations:** ^1^College of Life Sciences, Laboratory Center of Life Sciences, Nanjing Agricultural University, Nanjing, China; ^2^National Key Laboratory of Crop Genetics and Germplasm Enhancement, Jiangsu Collaborative Innovation Center for Modern Crop Production, Nanjing Agricultural University, Nanjing, China; ^3^Ministry of Agriculture’s Key Laboratory of Cotton and Rapeseed, Institute of Industrial Crops, Jiangsu Academy of Agricultural Sciences, Nanjing, China; ^4^College of Life Sciences, Shanxi Agricultural University, Taigu, China

**Keywords:** *Brassica napus* L., heterosis, nitric oxide, proteomics, salt stress

## Abstract

Heterosis is most frequently manifested as the superior performance of a hybrid than either of the parents, especially under stress conditions. Nitric oxide (NO) is a well-known gaseous signaling molecule that acts as a functional component during plant growth, development, and defense responses. In this study, the *Brassica napus* L. hybrid (F1, NJ4375 × MB1942) showed significant heterosis under salt stress, during both germination and post-germination periods. These phenotypes in the hybrid were in parallel with the better performance in redox homeostasis, including alleviation of reactive oxygen species accumulation and lipid peroxidation, and ion homeostasis, evaluated as a lower Na/K ratio in the leaves than parental lines. Meanwhile, stimulation of endogenous NO was more pronounced in hybrid plants, compared with parental lines, which might be mediated by nitrate reductase. Proteomic and biochemical analyses further revealed that protein abundance related to several metabolic processes, including chlorophyll biosynthesis, proline metabolism, and tricarboxylic acid cycle metabolism pathway, was greatly suppressed by salt stress in the two parental lines than in the hybrid. The above responses in hybrid plants were intensified by a NO-releasing compound, but abolished by a NO scavenger, both of which were matched with the changes in chlorophyll and proline contents. It was deduced that the above metabolic processes might play important roles in heterosis upon salt stress. Taken together, we proposed that heterosis derived from F1 hybridization in salt stress tolerance might be mediated by NO-dependent activation of defense responses and metabolic processes.

## Introduction

Salt stress is major environmental stress factor, which constrains plant growth and development and seriously limits agricultural worldwide production (Munné-Bosch et al., 2013; Zhao et al., 2020). On the basis of published evidence, about 20–50% arable land is impacted by salt stress, resulting in lower crop yields from saline-growing conditions (Bai et al., 2011; Farooq et al., 2020). Therefore, effective strategies for plants to tolerate salt stress are important so that agricultural production can be potentially recovered. It is well documented that salt stress influences many metabolic pathways, including carbohydrate metabolism, energy and amino acid metabolism, and photosynthesis, thereby influencing various physiological processes as well as plant growth and development (Xie et al., 2011; Nam et al., 2012; Prasch and Sonnewald, 2015; Duan et al., 2016). Most importantly, the accumulation of reactive oxygen species (ROS) elicited by salt stress can trigger oxidative stress in plants, which usually has detrimental impacts on cellular structures and macromolecules, including lipids, enzymes, and DNA (Miller et al., 2010; Chen et al., 2017; Ageeva-Kieferle et al., 2019; Zhao et al., 2020). Besides, salt toxicity is usually associated with increasing Na^+^ accumulation and inhibiting K^+^ uptake (Chen et al., 2017; Zhao et al., 2020). In order to cope with salt stress, various mechanisms were evolved to modulate ionic homeostasis. For example, the SOS pathway is in charge of Na^+^ transportation, thus initiating the transport of Na^+^ out of the cell and leading to the sequestration of Na^+^ in the vacuole (Zhu, 2003; Zhao et al., 2020).

One strategy toward enhancing plant salinity tolerance is to breed new varieties with built-in physiological processes to better cope with salinity. One such strategy is to develop hybrid crops (Flowers and Yeo, 1995; Flowers, 2004). It is well known that hybrid crops, such as maize and rice, have superior yield performance compared with their parental lines, a phenomenon commonly defined as heterosis or hybrid vigor (Lippman and Zamir, 2007; Miller et al., 2015). In plants, heterosis is a multigenic complex trait. It is understood to be the sum of multiple physiological and phenotypic traits, including changing flowering time and rate of botanic growth, increased biomass accumulation and yield, and resistance to biotic and abiotic environmental stresses (Lippman and Zamir, 2007; Baranwal et al., 2012; Ko et al., 2016; Feys et al., 2018). Importantly, heterosis is observed when plants are subjected to salt stress (Pérez-Alfocea et al., 1994; Xu et al., 2018). However, the traditional dominance and overdominance genetic models do not sufficiently explain the entire spectrum of growth and stress tolerance characteristics of heterosis in crop plants (Lippman and Zamir, 2007; Birchler et al., 2010; Krieger et al., 2010; Goff, 2011; Schnable and Springer, 2013). Therefore, it is urgent to elucidate related molecular mechanism of heterosis from a new perspective.

Nitric oxide (NO) is a cellular gaseous signaling molecule with multiple physiological functions during plant growth and stress responses (Lamattina et al., 2003; Bai et al., 2011; Wang, 2014; Zhang et al., 2018). It is well known that plants have several different enzymatic pathways to produce NO, and the most intensive investigation is based on nitrate reductase (NR) and a putative nitric oxide synthase (NOS) enzyme (Besson-Bard et al., 2008). Related investigations aiming to understand the signaling role of NO in plant tolerance against environmental stimuli have been accumulating (Zhao et al., 2007; Zheng et al., 2009; Bai et al., 2011). It is well documented that endogenous NO acting as a broad spectrum antistress molecule (Lamattina et al., 2003; Hasanuzzaman et al., 2018) could mediate various plant tolerance against oxidative stresses produced by UV-B (Tossi et al., 2009), drought (García-Mata and Lamattina, 2001), metal stress (Sun et al., 2014), and salinity (Wang et al., 2012; Zhao et al., 2004). Thus, NO orchestrates a wide range of processes in stressed plants. As a mechanism for plant tolerance against salinity stress, NO plays roles in alleviating germination inhibition (Zhao et al., 2007; Zheng et al., 2009), maintaining ion homeostasis (Zhao et al., 2004), re-establishing redox balance, and improving growth inhibition (Xie et al., 2013; Ageeva-Kieferle et al., 2019).

Proteomic technology is one of the potent approaches, which defines and describes the differential abundance proteins (DAPs) between the hybrid and its parents (Marcon et al., 2010; Nam et al., 2012). This approach could identify protein expression differences upon stress conditions (Bai et al., 2011; Duan et al., 2016; Dai et al., 2017). Numerous proteins have been identified that were modulated by salt stress and were involved in multiple metabolic processes. These include protein processing/turnover, osmolyte accumulation, carbon and energy metabolism, cytoprotection against oxidative damage, etc. (Bai et al., 2011; Nam et al., 2012). Considering the limitations of gel-separation and label-based proteomic techniques, a label-free quantitative approach is appropriate because it is more accurate, economical, and time-saving (Patel et al., 2009; Zhu et al., 2010).

*Brassica napus* L. is one of the most important oil crops worldwide because of its considerable economic and nutritional values (Jia et al., 2015). It is well known that the growth of *B. napus* seedlings was significantly inhibited by salt stress (Steppuhn et al., 2001; Jia et al., 2015; Zhao et al., 2018). Therefore, breeding new hybrid crops is one of the most effective strategies to promote salinity tolerance of *B. napus* plants. A recent report showed the important role of NO in hybrid chickpea during the germination period without environmental stress (Pandey et al., 2019). The aims of this study were to cultivate new varieties with better salinity tolerance in the near future, and most importantly, explain the molecular mechanism in heterosis of salinity tolerance: the involvement of NO signaling. The results of this study therefore not only assist in understanding the salt-adaptive mechanisms of hybrid plants, but also provide new ideas for agricultural production.

## Materials and Methods

### Plant Materials and Treatments

The two semi-dwarf male sterile lines and corresponding maintainers (M1942A/B, M1894A/B) derived from Polima sterile cytoplasm in *B. napus* L. were crossed by hand pollination with the restorer line NJ4375, to generate hybrids (F1, M1942 × NJ4375 and M1894 × NJ4375). These materials used in our experiments were developed and deposited at Nanjing Agriculture University. Considering that the M1942 and M1894 exhibited similar physiological properties and hybrid vigor, MB1942 (corresponding maintainer of M1942), NJ4375, and the generated hybrids (F1, MB1942 × NJ4375) were used.

The above three *B. napus* materials were grown in greenhouse pots filled with a mixture of vermiculite and quartz sand (3:1, v/v) and were irrigated with 1/2 Hoagland nutrient solution every 2 days. Six-week-old seedlings were cultured in 1/2 Hoagland nutrient solution with 200 mM NaCl or without NaCl (control group, Con) for another 14 days.

To evaluate the role of NO, 6-week-old seedlings of the above materials (NJ4375, MB1942, and F1) were pre-treated with sodium nitroprusside (SNP) (a well-known NO-releasing compound), 2-(4-carboxyphenyl)-4,4,5,5-tetramethylimidazoline-1-oxyl-3-oxide potassium salt (cPTIO, a fairly special scavenger of NO), or the control nutrient solution for 12 h. According to a previous report (Dong et al., 2015) and our pilot experiments, the concentrations of SNP and cPTIO were both used as 100 μM. After pre-treatments, the plants were transferred to 1/2 Hoagland nutrient solution with or without 200 mM NaCl for another 14 days. There were 18 experimental groups.

The roots and third leaves, harvested from different treatment plants at the indicated time points, were used immediately or frozen in liquid nitrogen and stored at −80°C for further analysis. At least three independent biological replicates were carried out for each treatment.

### Tolerance and Phenotype Analysis

For salt tolerance analysis, healthy seeds of each material in Petri dishes were germinated under darkness on distilled water with or without 200 mM NaCl for 3 days. Seeds were regarded as germinated when the radicle was equal to the length of the seeds.

Alternatively, 6-week-old seedlings of each material were irrigated with 1/2 Hoagland solution in the presence or absence of 200 mM NaCl for another 14 days. After treatments, representative growth images were recorded at the germination and post-germination periods. Meanwhile, the related phenotype observations, including germination rate, plant height, relative water content, biomass, and the contents of chlorophyll and proline, were then recorded (Xie et al., 2013; Su et al., 2018).

### Oxidative Damage Assay

After treatments, the third leaves were harvested from the plants. To detect lipid peroxidation, the concentration of thiobarbituric acid reactive substances (TBARS) was measured (Dai et al., 2017). In brief, approximately 0.5 g of leaf tissue was homogenized with 5 ml solution containing 0.25% 2-thiobarbituric acid (TBA) and 10% trichloroacetic acid (TCA). After heating and centrifugation, the absorbance at 440, 532, and 600 nm was recorded. The results were expressed as nmol g^–1^ fresh weight (FW).

For the determination of H_2_O_2_ and O_2_^-^, diaminobenzidine (DAB) and nitroblue tetrazolium (NBT) staining were used, respectively (Duan et al., 2016). To visualize H_2_O_2_ accumulation, the excised leaves were immersed in freshly prepared DAB solution (1 mg ml^–1^ DAB in 50 mM Tris–acetate buffer, pH 5.0), and then incubated in darkness at 25°C for 24 h. To visualize O_2_^-^ generation in the leaves, the excised tissue was stained with NBT solution (0.5 mg ml^–1^ NBT in 10 mM potassium phosphate buffer, pH 7.6), and then incubated in darkness at 25°C for 6 h. Afterward, the stained leaves were immersed in 95% (v/v) ethanol till chlorophyll was removed and then photographed.

### Measurement of Antioxidant Enzymes

Catalase (CAT) (EC 1.11.1.6) activity was analyzed by monitoring the consumption of H_2_O_2_ (ε = 39.4 mM^–1^ cm^–1^) at 240 nm for at least 3 min. Superoxide dismutase (SOD) (EC 1.15.1.1) activity was assessed by its capacity to inhibit NBT photochemical reduction that was determined at 560 nm (Su et al., 2019).

### Determination of Ion Content

According to previous reports (Chen et al., 2017), the ion content in the leaves was detected by using an inductively coupled plasma optical emission spectrometer (ICP-OES; Perkin Elmer Optima 2100 DV, PerkinElmer, Shelton, CT, United States). After treatment, fresh leaves were harvested and washed three times by distilled water. Afterward, oven-dried samples were digested by the Digital Block Sample Digestion System (ED54, LabTech, Beijing, China) with 2 ml HNO_3_. Na and K element contents were detected.

### Protein Extraction and Digestion

For total protein extraction in leaf tissues after treatments for 3 days, the Plant Total Protein Extraction Kit (Sigma-Aldrich, St. Louis, MO, United States) was used. Protein concentration was also quantified (Bradford, 1976).

Protein digestion was performed following a protocol defined previously (Wiśniewski et al., 2009). The trypsin-cleaved samples were desalted with C18 column (Thermo Fisher Scientific, Wilmington, MA, United States) and quantified by NanoDrop 2000 spectrophotometer (Thermo Fisher Scientific, Wilmington, MA, United States). Finally, the samples were freeze-dried before sample injection.

### Liquid Chromatography-Tandem Mass Spectrometry/Mass Spectrometry-Based Label Free Quantification and Date Analysis

For liquid chromatography-tandem mass spectrometry/mass spectrometry (LC-MS/MS) conditions, the label-free quantitative method was used to detect peptides (Duan et al., 2016). Three biological replicates for each of the different treatment groups were analyzed. A LTQ-Orbitrap mass spectrometer (Thermo Electron, Bremen, Germany) coupled to an Ultimate 3000 RSLC nano system (Dionex Thermo Fisher Scientific, Wilmington, MA, United States) was used for peptide analysis. The resulting peptides (2 μg each) were trapped on the trap column (Acclaim PepMap100 C18, 75 μm × 2 cm, 3 μm, 100 Å; Thermo Fisher Scientific, Wilmington, MA, United States) at a flow rate of 4 μl min^–1^ in loading buffer (2% acetonitrile, 0.1% formic acid in HPLC-grade water) for 15 min, followed by separation on an analytical column (Acclaim PepMap^§^ RSLC, C18, 75 μm × 15 cm, 3 μm, 100 Å, Thermo Fisher Scientific, Wilmington, MA, United States) in a linear gradient from 3 to 45% of solvent B (80% acetonitrile and 0.1% FA) at a flow rate of 0.25 μl min^–1^ over 112 min. The mass spectrometer was operated with an electrospray voltage of 2.3 kV. From the 60,000 resolution MS^1^ full scan in the range 350–1,800 *m*/*z*, the top five most prominent ions were selected for MS/MS analysis if they exceeded intensity greater than 5,000 counts and if they were at least doubly charged. The normalized collision energy for HCD was set to a value of 40%, and the resulting fragments were detected with 7,500 resolution in the Orbitrap. Every selected ion was dynamically excluded from further MS/MS fragmentation for 60 s.

Raw data were analyzed using the Proteome Discoverer Software (version 1.4, Thermo Fisher Scientific, Waltham, MA, United States) against UniProtKB^1^, which contained a database of accessible *Arabidopsis* and *Brassica* protein sequences. Searching parameters were as follows: at most two missed trypsin cleavage allowed, MS tolerance of 10 ppm, cysteine treated by iodoacetamide, and oxidation of methionine. A false discovery rate (FDR) based on *q* value was estimated, and only peptides at the 99% confidence interval were counted as the identified protein. Relative quantitation for proteins between the control and treated groups was obtained and analyzed. Proteins with a | fold change (FC)| ≥ 1.5 are considered to be a DAP in this study.

### Bioinformatics Analysis

In this study, bioinformatics analysis of the obtained DAPs was conducted through OmicsBean (a multiomics data analysis tool)^2^, which integrated gene ontology (GO) and Kyoto Encyclopedia of Gene and Genomes (KEGG) pathways. The co-existing DAPs among different comparing pairs were located by using Venn diagram online tool^3^. All proteins identified in the *B. napus* proteome were based on their corresponding homologs in the *Arabidopsis thaliana* proteome database by using the *B. napus* Genome Browser^4^.

### NO Assay

The content of NO was monitored by laser scanning confocal microscopy (LSCM) with a fairly specific NO fluorescent probe, 4-amino-5-methylamino-2′,7′-difluorofluorescein diacetate (DAF-FM DA) (Li et al., 2012). At least five individual samples were randomly selected and measured per treatment. The fluorescence of NO was quantified as relative fluorescence units using the Volocity Demo software 2.5 (PerkinElmer, Waltham, MA, United States).

### RNA Extraction and Gene Expression Analysis

Total RNA was extracted by TransZol Up Kit (TransGen Biotech, Beijing, China) according to the manufacturer’s instructions. DNA-free total RNA was then reverse-transcribed into cDNA by oligo(dT) primers and SuperScript reverse transcriptase (Invitrogen, Carlsbad, CA, United States). The quantitative real-time PCR (qPCR) was performed by using Mastercycler^§^ ep *realplex* real-time PCR system (Eppendorf, Hamburg, Germany) with TransStart Top Green qPCR SuperMix (TransGen Biotech, Beijing, China). The sequences of the primers are given in Supplementary Table 1. The relative expression levels of the corresponding genes were presented as values compared with the corresponding control samples at the indicated conditions or lines, after normalization with two reference genes *BnActin* and *BnGAPDH*. The relative gene expression levels were analyzed by using the 2^–ΔΔ CT^ method (Livak and Schmittgen, 2001).

### Statistical Analysis

All results expressed were the mean values ± standard deviation (SD) of at least three independent experiments with three replicates each. For statistical analysis, differences among treatments were analyzed by one-way analysis of variance (ANOVA) (*P* < 0.05, Duncan’s multiple range test) or two-way ANOVA (*P* < 0.05, Student’s *t* test).

## Results

### Heterosis in Salt Stress Tolerance

**FIGURE 1:**
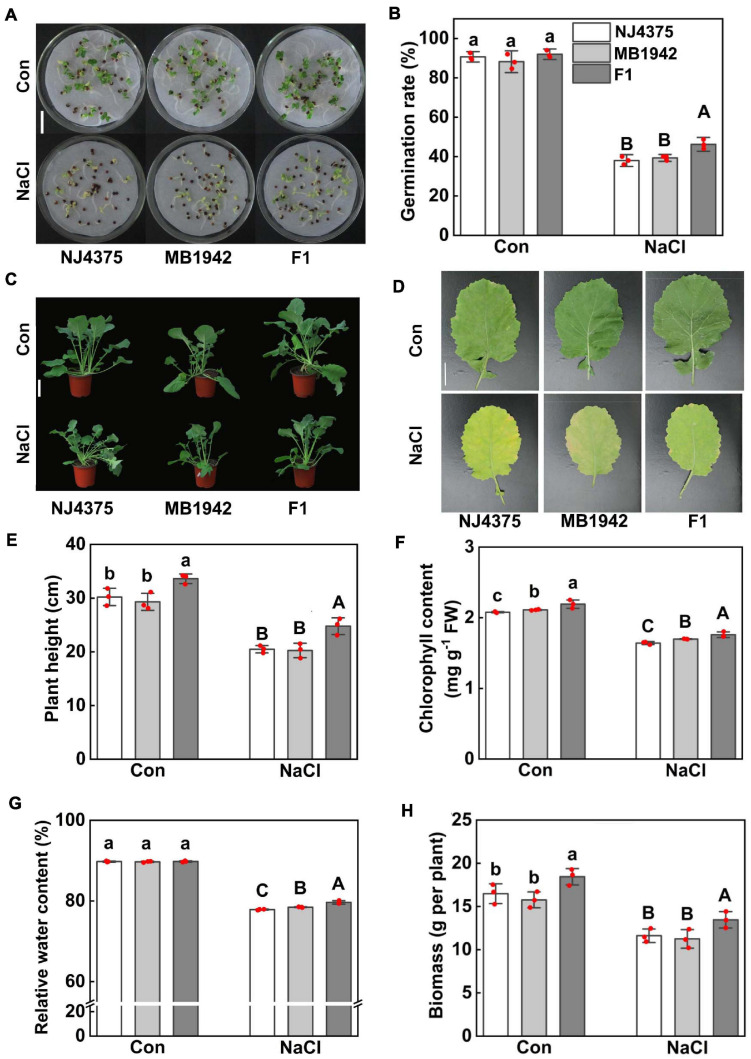
Heterosis of *B. napus* hybrid (F1) and their parental lines (NJ4375 and MB1942) on the phenotypic manifestation and salt tolerance assays during germination and post-germination. **(A,B)** Germination stage. Bar = 3 cm. **(C,D)** Post-germination stage. Bar = 10 cm **(C)**. The leaves were sampled and photographed. Bar = 3 cm **(D)**. The plant height **(E)**, chlorophyll content **(F)**, relative water content **(G)**, and biomass **(H)** were determined. Treatment with distilled water **(A,B)** or 1/2 Hoagland nutrient solution **(C–H)** was regarded as control (Con). Values are the means ± SD for three independent experiments. One-way ANOVA analyses were conducted according to Duncan’s multiple range test. Within each set of experiments, bars with different letters denote significant differences at *P* < 0.05.

During the germination period, the hybrids showed significant higher germination rate than its parents in the presence of NaCl (NJ4375 and MB1942; Figures 1A,B). In the post-germination stage, the hybrid rapeseed plants grew taller, and the mature hybrid leaves were greener than its parents (Figures 1C,D). For the mean trait values, it was observed that NJ4375 and MB1942 were more sensitive to salinity stress than hybrid plants, evaluated by changes in plant height (decreasing to 32.2, 30.9, and 26.4% of the non-stressed controls, respectively; Figure 1E), chlorophyll content (20.7, 20.0, and 19.3%, respectively; Figure 1F), relative water content (13.3, 12.55, and 11.3%, respectively; Figure 1G), and biomass (29.5, 28.61, and 27.1%, respectively; Figure 1H). Together, rapeseed heterosis in salt stress tolerance is confirmed.

### Heterosis in Alleviating Oxidative Damage and Maintaining Ion Homeostasis

**FIGURE 2:**
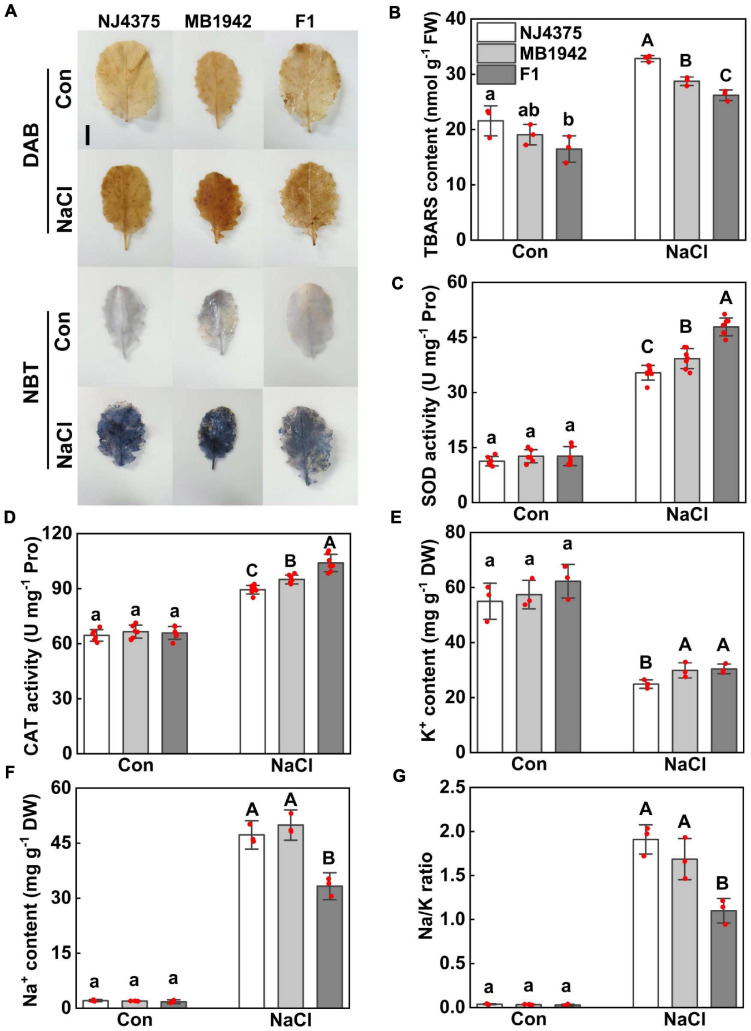
Heterosis of *B. napus* hybrid (F1) and their parental lines (NJ4375 and MB1942) on alleviating oxidative damage and maintaining ion homeostasis. **(A)** Leaves were collected and stained with 3,3′-diaminobenzidine (DAB) or nitroblue tetrazolium (NBT) to visualize H_2_O_2_ or O_2_^-^ distribution, respectively. Bar = 3 cm. Meanwhile, TBARS content **(B)**, SOD activity **(C)**, CAT activity **(D)**, K^+^ **(E)**, and Na^+^ **(F)** contents, as well as Na/K ratio **(G)** in leaf tissues, were measured. Treatment with distilled water was regarded as control (Con). Values are the means ± SD for three or five independent experiments. One-way ANOVA analyses were conducted according to Duncan’s multiple range test. Within each set of experiments, bars with different letters denote significant differences at *P* < 0.05.

Salt stress-induced oxidative stress in plants is usually brought about redox imbalance (Zhu, 2016; Chen et al., 2017; Zhao et al., 2020). In order to investigate the effect of heterosis on alleviating salt stress-induced oxidative damage, histochemical staining and antioxidant enzyme activities were analyzed. The levels of H_2_O_2_ and O_2_^-^ were respectively monitored by DAB staining and NBT staining. The two parental lines, when stressed by NaCl, were stained extensively in the leaves, in comparison with chemical-free control samples (Figure 2A). However, the accumulation of H_2_O_2_ and O_2_^-^ was partially reduced in hybrids. Meanwhile, changes in TBARS levels were observed to exhibit a comparable pattern (Figure 2B). The activities of two antioxidant enzymes, namely SOD and CAT, were also measured and compared (Figures 2C,D). The results showed that when challenged with NaCl stress, the enhanced enzyme activities were more pronounced in the hybrid than those in the parental lines. The above results clearly suggest that hybrids could better alleviate salt stress-induced oxidative damage.

Additionally, it has been well-documented that ionic balance inside cells is closely related to plant tolerance against salt stress (Zhu, 2003; Wu et al., 2020; Zhao et al., 2020). Compared with parental lines, a lower Na/K ratio in hybrid plants was observed in response to salt stress conditions, which was supported by the results, showing a higher K^+^ level and a significant lower Na^+^ content (especially) in the seedlings (Figures 2E–G).

### Global Characterization of Protein Expression Patterns

**FIGURE 3:**
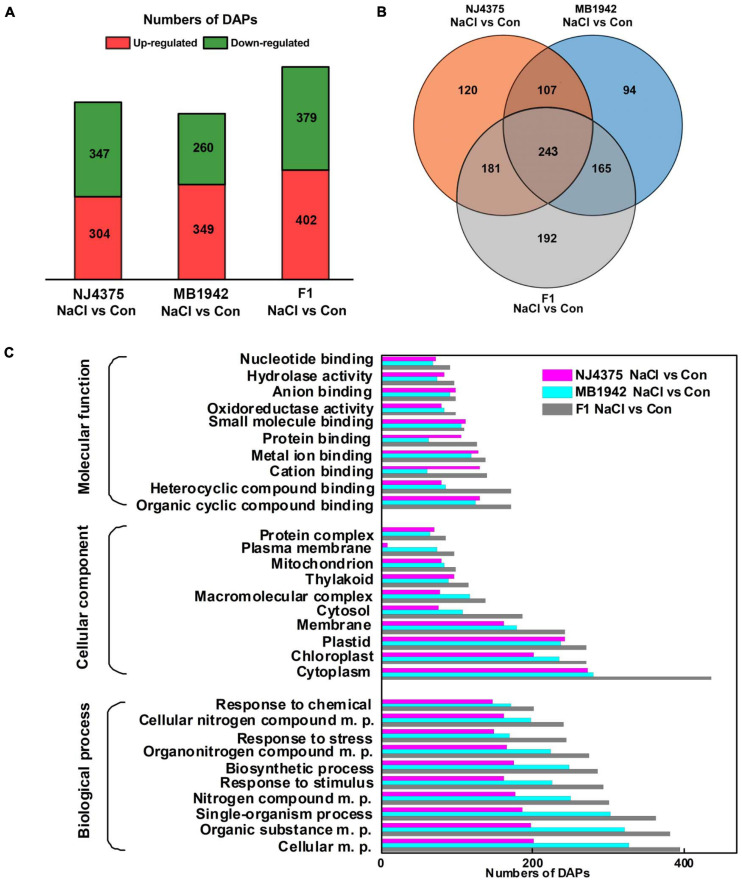
Bioinformatics analysis of identified differential abundance proteins (DAPs). Treatment with distilled water was regarded as control (Con). Venn diagram of DAPs identified in hybrid and its parental lines **(A)**. The numbers of up- and down-regulated proteins were given **(B)**. Gene ontology (GO) **(C)** classification of DAPs was collected and presented. The plots reveal the distribution of the 10 most significantly enriched terms.

Although some knowledge has been published regarding understanding of plant salt adaptation (Zhu, 2003; Xie et al., 2013; Wu et al., 2020), specific mechanisms aligned with heterosis for plant salt tolerance remain ambiguous. In order to obtain an overall view of proteomic changes of the three *B. napus* materials under salt stress, comparative proteomic analysis was performed by LC-MS/MS-based label-free quantification. Compared with control conditions, approximately 1,500 proteins (NaCl stress/control) were identified in the stressed leaves of the three materials (NJ4375, MB1942, or F1; Supplementary Table 2). Applying a threshold of minimum FC with a cutoff at 1.5-fold (up-regulation) and 0.67-fold (downregulation), a total of 651 (347 up-regulated and 304 downregulated), 609 (260 up-regulated and 349 downregulated), and 781 (379 up-regulated and 402 downregulated) proteins showed differential accumulation in NaCl-treated NJ4375, MB1942, and F1 groups, respectively (Figure 3A). By using the Venn diagram to analyze total DAPs among the three NaCl-treated materials, 120, 94, and 192 specific DAPs were found in NJ4375, MB1942, and F1, respectively. Meanwhile, there were 243 overlapping DAPs among the three *B. napus* genotypes (Figure 3B).

Gene ontology term analysis for biological processes was carried out to ascertain functional annotations of DAP clusters, with the top 10 significantly enriched terms shown in Figure 3C. In all three major functions, namely biological process (BP), cellular component (CC), and molecular function (MF), the hybrid showed better adaptation than its parental lines for most functions. In BP analysis, for response to stimulus, response to stress, and response to chemical, these three representative terms showed that there were more DAP responses to NaCl-induced stress conditions. The CC analysis showed that most DAPs are related to stress response belonging to the cytoplasm, chloroplast, plastid, membrane, and cytosol. Heterocyclic compound binding and organic cyclic compound binding were the dominant MF in GO assignments.

### Endogenous NO Was Involved in the Heterosis of *B. napus* Upon Salt Stress

**FIGURE 4:**
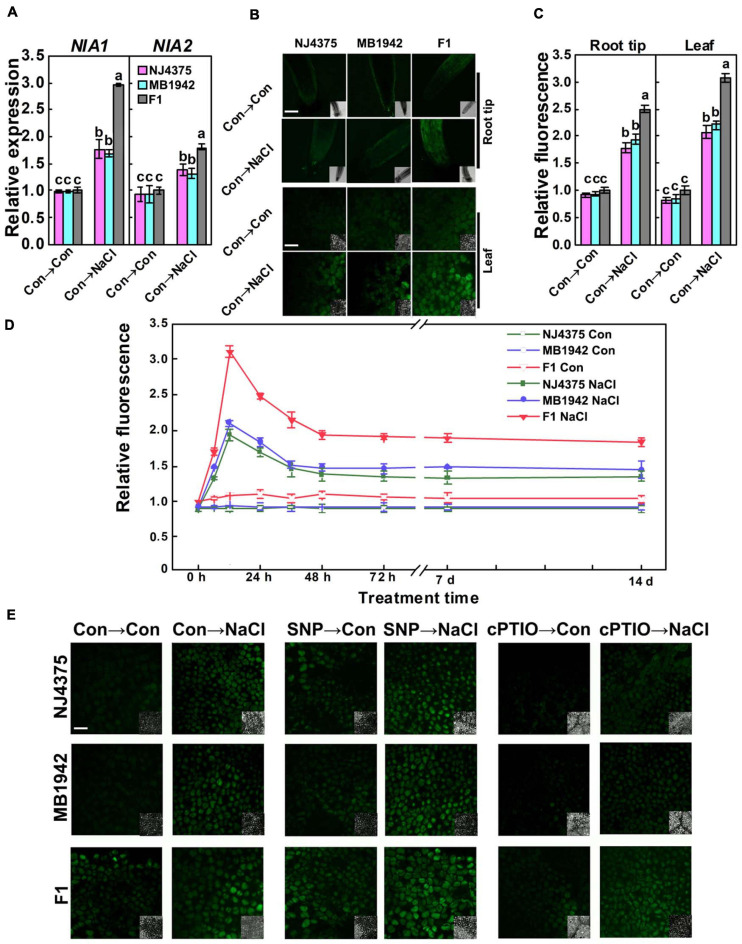
Changes in nitric oxide (NO) homeostasis. **(A)** The gene expression levels of *NIA1* and *NIA2* in the leaves with or without stress for 6 h were analyzed by qPCR. **(B)** After treatment for 12 h, root tips and leaves were loaded with 4-amino-5-methylamino-2′,7′-difluorofluorescein diacetate (DAF-FM DA) and detected by laser scanning confocal microscopy (LSCM). DAF-FM DA-dependent fluorescence densities according to panel **(B)** were given panel **(C)**. The time course of NO-related fluorescence in the leaves was also shown panel **(D)**. After treated for 12 h, the NO levels in the leaves were detected by LSCM **(E)** with or without SNP or cPTIO pre-treatment, respectively. Values are the means ± SD for three independent experiments. One-way ANOVA analyses were conducted according to Duncan’s multiple range test. Different letters denote significant differences at *P* < 0.05.

Interestingly, proteomic data also showed that NO might be involved in the heterotic responses of *B. napus* under salt stress. The expression levels of two identified proteins (NIA1 particularly and NIA2, accession: P39867 and P39868, two key proteins of NO metabolism in *B. napus*) of the hybrid were higher than those in NJ4375 and MB1942 (Table 1). Gene expression levels (*BnNIA1* especially and *BnNIA2*) showed a similar pattern (Figure 4A).

**TABLE 1:** List of NO metabolism proteins in the three *B. napus* materials/seedling leaves in response to salt stress for 3 days.

Accession	Description	Gene name	Ratio				
			NJ4375 Con/F1 Con	MB1942 Con/F1 Con	NJ4375 NaCl/F1 Con	MB1942 NaCl/F1 Con	F1 NaCl/F1 Con
P39867	Nitrate reductase 1	*NIA1*	0.997	0.961	1.908	1.781	2.812
P39868	Nitrate reductase 2	*NIA2*	0.954	0.959	1.380	1.263	1.843

In order to further explore the mechanisms of heterosis control of salt stress tolerance, NO production in root and leaf tissues of two parental lines and hybrid plants was observed by labeling NO using its fairly specific fluorescence probe DAF-FM DA, which can be used widely to monitor NO levels in plant cells (Figure 4B; Foresi et al., 2010). By using LSCM, the NO-related signal was captured as green fluorescence. Under control and salt stress (in particularly) conditions, the hybrid both showed higher DAF-FM-dependent fluorescence, in the root tips and leaves, compared with parental lines (12 h; Figure 4B). Meanwhile, DAF-FM-dependent fluorescence displayed higher intensity in the leaves than in the root tips (Figure 4C). In addition, the NO levels of plants during the germination stage were also detected. The results showed that NO level in the hybrid was significantly higher than its parental lines under salt stress (in particular) and non-stressed conditions (Supplementary Figure 1). These results indicated the possible roles of NO at both germination and post-germination stages.

The time-course imaging in the leaves of the three materials is shown in Figure 4D. Images showed strong and substantial increased fluorescence in the three plant materials, respectively, all peaking at 12 h, followed by a mild decrease until 14 days after NaCl treatment, in comparison with the basal levels in the control samples. We also noticed that the response of hybrids is more pronounced than those in NJ4375 and MB1942, two parental lines.

As shown in Figure 4E, since the above DAF-FM-dependent fluorescence intensity in the seedling leaves of the three materials was respectively impaired by pre-treatment with cPTIO (a scavenger of NO) and the fluorescence was markedly intensified in the presence of a well-known NO-releasing compound SNP (a positive control), we further confirmed that the DAF-FM-dependent fluorescence is mainly caused by endogenous NO levels. Combined with the phenotypic parameters in the presence of NaCl stress (Figure 1), it was further deduced that there is potential connection between NO and heterosis under salt stress.

### NO Was Involved in Chlorophyll Biosynthesis in the Hybrid

**FIGURE 5:**
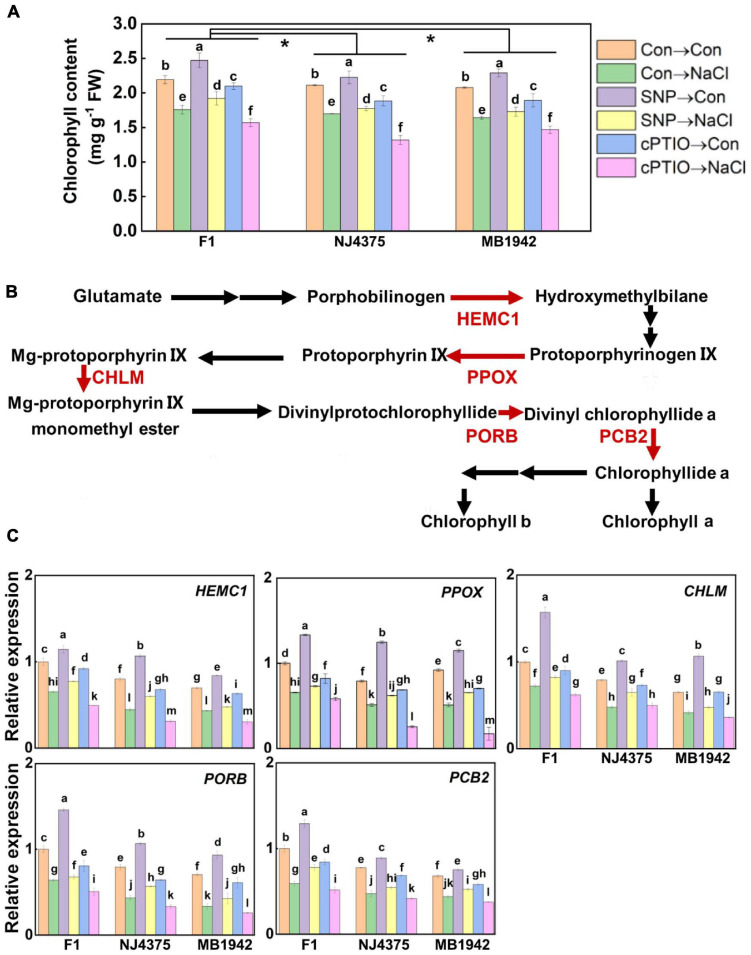
The role of NO in the regulated chlorophyll metabolic process. **(A)** Chlorophyll content was detected after treatment for 14 days. **(B)** After stress for 3 days, the proteins were extracted from the leaf tissues. Chlorophyll metabolism-related proteins were identified by LC-MS/MS. Compared with the parental lines, the less pronounced decreased proteins associated with NO in the hybrid, including hydroxymethylbilane synthase (HEMC1), protoporphyrinogen/coproporphyrinogen III oxidase, (PPOX), magnesium-protoporphyrin *O*-methyltransferase (CHLM), protochlorophyllide reductase (PORB), and divinyl chlorophyllide-a 8-vinyl-reductase (PCB2), are shown in red (data obtained from Supplementary Table 2). **(C)** After treated for 1 day, the expression profiles of the corresponding genes in the leaves of F1 and two parents were analyzed by qPCR. Values are the means ± SD for three independent experiments. For panel **(A)**, bars with different letters represent significant differences among treatments in a genotype (one-way ANOVA, *P* < 0.05, Duncan’s multiple range test), and the asterisk indicates significant difference between genotype × treatment interactions (two-way ANOVA, *P* < 0.05, Student’s *t* test). For panel **(C)**, bars with different letters represent significant differences (one-way ANOVA, *P* < 0.05, Duncan’s multiple range test).

Chlorophyll synthesis is strongly linked to photosynthetic activity (Fujimoto et al., 2012; Wang and Grimm, 2021). Under salt stress, chlorophyll biosynthesis was substantially downregulated in two parent materials (particularly in MB1942) and hybrid plants (Figure 5A; Chen et al., 2017). In the presence of SNP with or without NaCl stress, chlorophyll contents were differentially increased in the above three materials, and contrasting tendencies were observed in the presence of cPTIO.

Our proteomic data further revealed that compared with those in hybrid plants subjected to NaCl stress, the decreased levels of the function proteins associated with chlorophyll biosynthesis were more pronounced in their parents (Table 2, Figure 5B, and Supplementary Table 2). These proteins include hydroxymethylbilane synthase (HEMC1, accession: A0A078G803), protoporphyrinogen/coproporphyrinogen III oxidase (PPOX, accession: A0A078FSF5), magnesium-protoporphyrin *O*-methyltransferase (CHLM, accession: A0A078HA16), protochlorophyllide reductase (PORB, accession: A0A078GUT8), and divinyl chlorophyllide-a 8-vinyl-reductase (PCB2, accession: A0A078FHJ5). However, the proteomic levels related to chlorophyll biosynthesis in hybrid seedlings were significantly enhanced under salt stress in the presence of SNP, but were obviously impaired by cPTIO pre-treatment (Table 2), all of which matched with the changes in chlorophyll contents (Figure 5A), reflecting the important role of NO. Similar phenotypic data in the changes of chlorophyll contents were observed in two parents, in the presence of either SNP or cPTIO. The above results were confirmed by the data obtained with qPCR (Figure 5C).

**TABLE 2:** List of chlorophyll metabolism proteins in the seedling leaves of *B. napus* materials in response to salt stress for 3 days with or without sodium nitroprusside (SNP) or 2-(4-carboxyphenyl)-4,4,5,5-tetramethylimidazoline-1-oxyl-3-oxide potassium salt (cPTIO) pre-treatment for 12 h.

Accession	Description	Gene name	Ratio upon NaCl treatment			
			NJ4375/F1	MB1942/F1	F1 SNP/Con	F1 cPTIO/Con
A0A078G803	Hydroxymethylbilane synthase	*HEMC1*	0.203	0.152	2.065	0.631
A0A078FSF5	Protoporphyrinogen/coproporphyrinogen III oxidase	*PPOX*	0.263	0.275	4.852	0.491
A0A078HA16	Magnesium-protoporphyrin *O*-methyltransferase	*CHLM*	0.206	0.250	2.088	0.572
A0A078GUT8	Protochlorophyllide reductase	*PORB*	0.489	0.477	46.212	0.451
A0A078FHJ5	Divinyl chlorophyllide-a 8-vinyl-reductase	*PCB2*	0.351	0.339	9.302	0.268

### Proline Metabolic Pathway in the Hybrid Was Dependent on NO

**FIGURE 6:**
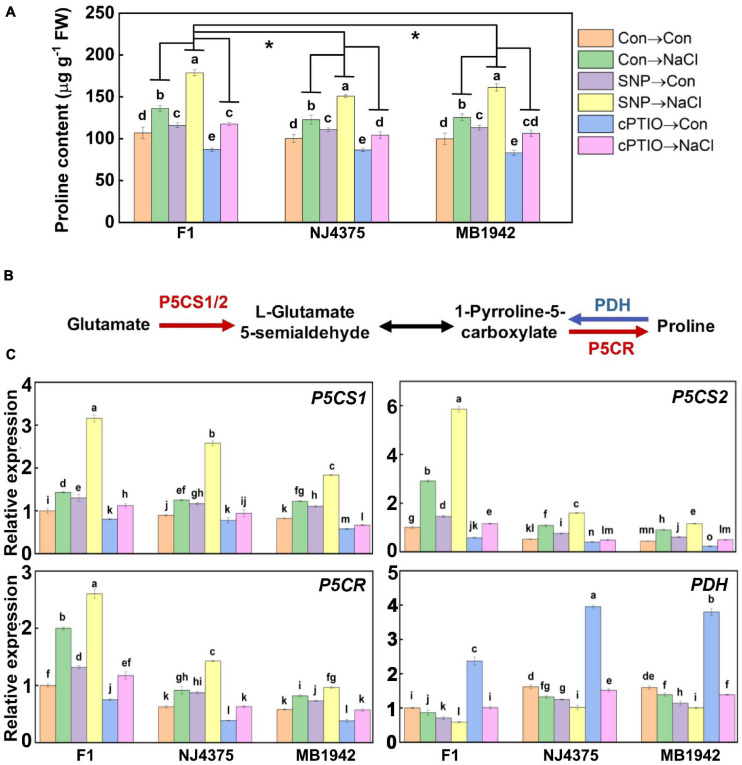
The involvement of NO in the regulated proline biosynthesis. **(A)** Proline content was detected after treatments for 14 days. **(B)** After treated for 3 days, the proteins were extracted from the leaf tissues. Proline biosynthesis-related proteins were identified by LC-MS/MS. Compared with the parental lines, the up-regulated proteins associated with NO in the hybrid, including delta-1-pyrroline-5-carboxylate synthase A (P5CS1), delta-1-pyrroline-5-carboxylate synthase B (P5CS2), and pyrroline-5-carboxylate reductase (P5CR), are shown in red, and the down-regulated protein [proline dehydrogenase (PDH)] is shown in blue (data obtained from Supplementary Table 2). **(C)** After treated for 1 day, the expression profiles of the corresponding genes in the leaves of F1 and two parents were analyzed by qPCR. Values are the means ± SD for three independent experiments. For panel **(A)**, bars with different letters represent significant differences among treatments in a genotype (one-way ANOVA, *P* < 0.05, Duncan’s multiple range test), and the asterisk indicates significant difference between genotype × treatment interactions (two-way ANOVA, *P* < 0.05, Student’s *t* test). For panel **(C)**, bars with different letters represent significant differences (one-way ANOVA, *P* < 0.05, Duncan’s multiple range test).

As shown in Figure 6A, there was no obvious difference in proline contents among the three *B. napus* materials under chemical-free control conditions. In the presence of NaCl stress, however, the enhanced proline content was pronounced in the hybrids compared with its parental lines. Importantly, these changes in the three stressed materials were further significantly intensified by SNP, but blocked by cPTIO. Also, cPTIO alone resulted in the reduction of proline content compared with the corresponding chemical-free controls.

Subsequently, several proteins involved in proline biosynthesis (Table 3, Figure 6B, and Supplementary Table 2), such as delta-1-pyrroline-5-carboxylate synthase A (P5CS1) (accession: A0A078E0Y2), delta-1-pyrroline-5-carboxylate synthase B (P5CS2) (accession: A0A078HWQ9), pyrroline-5-carboxylate reductase (P5CR) (accession: A0A078F9Z9), and proline dehydrogenase (PDH) (accession: A0A078G092) in *B. napus* seedlings, were analyzed. In plants, proline is mainly produced from glutamate. In this pathway, the enzymes P5CS1 and P5CS2 reduce glutamate to L-glutamate 5-semialdehyde, and the P5CR reduces 1-pyrroline-5-carboxylate to proline (Szabados and Savouré, 2010). Conversely, the enzyme PDH results in proline breakdown and converts proline to 1-pyrroline-5-carboxylate (Ribarits et al., 2007). As expected, the corresponding genes encoding three enzymes (P5CS1, P5CS2, and P5CR) required for proline synthesis and related protein levels in the hybrids exhibited higher abundance than those in their parents upon salt stress (Figures 6B,C and Table 3). In contrast, the abundance of the *PDH* transcript, encoding an enzyme for proline degradation, and its protein levels were lower in the hybrid under identical conditions.

**TABLE 3:** List of proline metabolism proteins in three seedling leaves of *B. napus* materials in response to salt stress for 3 days with or without SNP or cPTIO pre-treatment for 12 h.

Accession	Description	Gene name	Ratio upon NaCl treatment			
			NJ4375/F1	MB1942/F1	F1 SNP/Con	F1 cPTIO/Con
A0A078E0Y2	Delta-1-pyrroline-5-carboxylate synthase A	*P5CS1*	0.334	0.396	3.422	0.401
A0A078HWQ9	Delta-1-pyrroline-5-carboxylate synthase B	*P5CS2*	0.439	0.445	1.815	0.552
A0A078F9Z9	Pyrroline-5-carboxylate reductase	*P5CR*	0.548	0.548	2.039	0.605
A0A078G092	Proline dehydrogenase	*PDH*	3.004	1.821	0.553	2.453

To investigate the link between NO and proline production, either SNP or cPTIO was separately used, in the presence or absence of NaCl stress. Results showed that exogenous SNP administration increased some protein levels in the stressed hybrid plants, including P5CS1, P5CS2, and P5CR, and decreased PDH level (Table 3). These results were confirmed by the changes in related transcripts (Figure 6C). Meanwhile, contrasting results were observed when cPTIO was applied together in hybrid materials, in both protein and transcriptional levels (Table 3 and Figure 6C).

### The Hybrid Regulated the Tricarboxylic Acid Cycle via Endogenous NO

**FIGURE 7:**
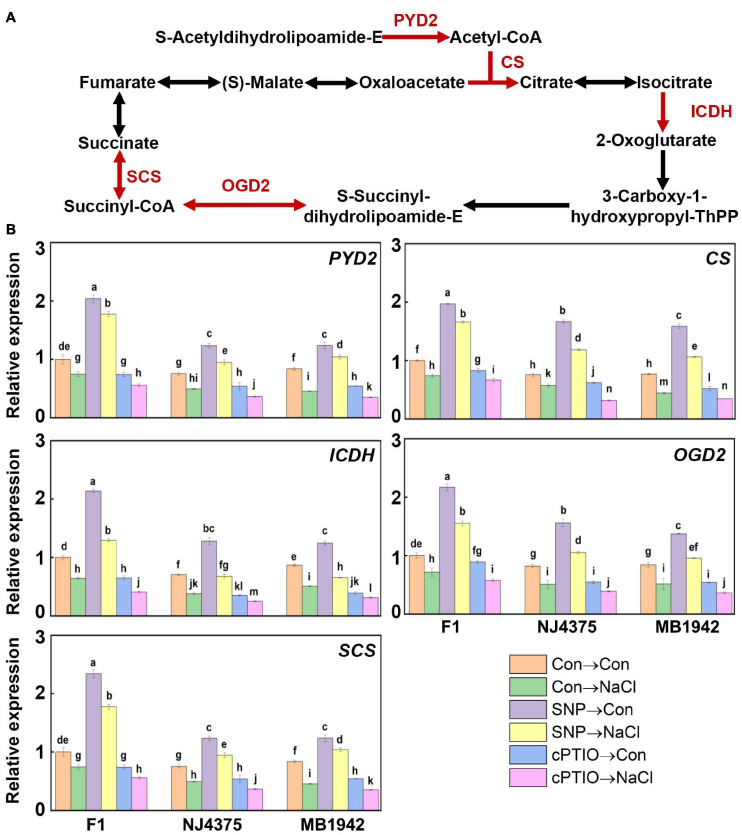
NO-modulated TCA cycle metabolic process. **(A)** The proteins were extracted from the leaf tissues after treated for 3 days. TCA cycle metabolic-related proteins were identified by LC-MS/MS. The up-regulated proteins, including dihydrolipoamide acetyltransferase (PYD2), citrate synthase (CS), isocitrate dehydrogenase (ICDH), dihydrolipoamide succinyltransferase (OGD2), and succinyl-CoA synthetase (SCS) associated with NO in the hybrid, are shown in red (data obtained from Supplementary Table 2). **(B)** After treated for 1 day, the expression profiles of the corresponding genes in the leaves of F1 and two parents were analyzed by qPCR. Values are the means ± SD for three independent experiments. One-way ANOVA analyses were conducted according to Duncan’s multiple range test. Different letters denote significant differences at *P* < 0.05.

It is well known that the TCA cycle-related enzymes are obviously sensitive to oxidative stimuli, and abiotic stress-triggered oxidative stress could significantly inhibit key enzymes of the TCA cycle (Morgan et al., 2008; Che-Othman et al., 2017). In this proteomic study, five key proteins of the TCA cycle, including dihydrolipoamide acetyltransferase (PYD2, accession: A0A078FMN3), citrate synthase (CS) (accession: A0A078IH04), isocitrate dehydrogenase (ICDH) (accession: A0A078FVI0), and succinyl-CoA synthetase (SCS) (accession: A0A078HSE9), in two parental lines were downregulated during salt stress (Table 4, Figure 7A, and Supplementary Table 2). Nevertheless, changes in dihydrolipoamide succinyltransferase (OGD2, accession: A0A078FRZ0) displayed increased/decreased tendency in NJ4375 and MB1942, respectively. However, heterosis helped the *B. napus* hybrids to counteract (in particular) or intensify (only OGD2 in NJ4375) the changes in the above key TCA cycle proteins caused by salt stress.

**TABLE 4:** List of TCA cycle-related proteins in the seedling leaves of *B. napus* materials in response to salt stress for 3 days with or without SNP or cPTIO pre-treatment for 12 h.

Accession	Description	Gene name	Ratio upon NaCl treatment			
			NJ4375/F1	MB1942/F1	F1 SNP/Con	F1 cPTIO/Con
A0A078FMN3	Dihydrolipoamide acetyltransferase	*PYD2*	0.227	0.199	3.775	0.589
A0A078IH04	Citrate synthase	*CS*	0.331	0.313	16.720	0.373
A0A078FVI0	Isocitrate dehydrogenase	*ICDH*	0.490	0.328	2.460	0.333
A0A078FRZ0	Dihydrolipoamide succinyltransferase	*OGD2*	0.528	0.436	1.865	0.491
A0A078HSE9	Succinyl-CoA synthetase	*SCS*	0.394	0.384	2.023	0.478

In order to further confirm the role of NO in the hybrid, SNP and cPTIO were used. The results showed that the protein levels related to TCA in heterosis were enhanced by SNP, but inhibited by cPTIO, indicating the central role of NO homeostasis (Table 4). This hypothesis above was obviously verified by qPCR data (Figure 7B).

## Discussion

To date, many reports observed that hybrid plants have advantages in terms of plant growth and tolerance of environmental stimuli. For example, increased leaf area of the hybrid plant *Arabidopsis* resulted in increased biomass (Fujimoto et al., 2012). Improved germination was observed in the hybrid plant of chickpea because of its reduced ROS level (Pandey et al., 2019). In addition, hybrids showed obvious heterosis for photosynthesis under salt stress (Xu et al., 2018). However, very little is known about the corresponding mechanisms regarding heterosis of salinity tolerance in plants.

The current study provides a main branch of molecular basis for heterosis, showing that hybrid plants’ tolerance against salt stress might require NO signaling, including activation of defense responses and metabolic processes. Since NO is previously regarded as a gaseous transmitter in prokaryotes and eukaryotes (Lamattina et al., 2003; Besson-Bard et al., 2008), the above results further reflected the importance of NO in plants, especially in heterosis.

In this study, we found that the hybrid of *B. napus* resulted in the promotion of seed germination and seedling growth under salt stress (Figures 1A–D), which were confirmed by the alleviation of reduction in plant height (Figure 1E), chlorophyll (Figure 1F), relative water content (Figure 1G), and biomass (Figure 1H). These results in *B. napus* were consistent with previous studies (Akram et al., 2010; Fujimoto et al., 2012; Bagum et al., 2017; Xu et al., 2018), showing that hybrid plants were more tolerant against salinity.

The imbalance of ROS elicited by salt stress usually brings about oxidative stress and cellular damage (Miller et al., 2010; Chen et al., 2017). In this study, it was observed that the improved tolerance in *B. napus* hybrids when challenged with salt stress was explained by alleviating oxidative damage, confirmed by the elimination of H_2_O_2_ and O_2_^-^ production (DAB and NBT staining, respectively), decreased TBARS content, and increased CAT and SOD activities (Figures 2A–D). Importantly, the ion homeostasis plays a critical role in enabling the plant to tolerate salt stress (Zhu, 2003; Zhao et al., 2004; Wu et al., 2020). Our work subsequently implicated that hybrid plants confer increased salt stress tolerance partly by maintaining ion homeostasis, showing a lower Na^+^/K^+^ ratio in the stressed hybrids than parental lines (Figures 2E–G). Further experiments regarding kinetic changes in K^+^ retention and Na^+^ exclusion and the corresponding mechanism related to NO signaling (Pérez-Alfocea et al., 1996; Zhao et al., 2004, 2007; Xu et al., 2018; Farooq et al., 2020) should be carried out in the near future.

To further probe molecular mechanism, a proteomic analysis was performed by LC-MS/MS and related data were compared among different treatments in the three *B. napus* materials/seedling leaves. Based on the criterion of | fold change (FC)| ≥ 1.5, 1,102 DAPs were found in three *B. napus* materials under NaCl treatment or control (Figures 3A,B and Supplementary Table 2). Further GO enrichment analysis revealed that more DAPs, for example, related to response to stress and organic substance in biological process, were highly enriched in F1 plants (Figure 3C). We also noticed differential profiles of stress-related potassium channel beta subunit 1 (KAB1; Sun et al., 2016) in both hybrids and parental lines upon salinity stress (Supplementary Table 2), which matched with the changes in Na^+^/K^+^ ratio (Figure 2G). The proteomic data also suggested that multiple mechanisms might be involved in heterosis-driven salt tolerance in *B. napus* plants.

Many investigations regarding the tolerance mechanisms underlying heterosis of salinity tolerance have been reported (Akram et al., 2010; Fujimoto et al., 2012; Bagum et al., 2017; Xu et al., 2018; Farooq et al., 2020). However, the role of NO in heterosis has been rarely considered. As an important gaseous signaling molecule, NO participates in multiple plant tolerance reactions when challenged with environmental stimuli (Lamattina et al., 2003; Wang, 2014; Sánchez-Vicente et al., 2019). The present study demonstrated that two essential NO synthetic proteins (NIA1 and NIA2) were significantly increased in the F1 hybrid in comparison with the parental lines, especially under salt stress (Table 1). The results above were parallel to the transcriptional profiles detected by qPCR (Figure 4A). In subsequent trials, NO accumulation in root and leaf tissues was also observed by LSCM. For example, in the strongly salt-tolerant hybrid plants, NO production was substantially increased in response to salt stress, in both root and leaf tissues (particularly Figures 4B,C). These results further confirmed that NaCl-triggered NO synthesis was due to the up-regulation of NR gene expression.

The production of stress-induced endogenous signaling molecules is generally time dependent and plays different roles in response to various stresses (Sun et al., 2014; Xie et al., 2014; Chen et al., 2017). During the 14-day treatment period, the time course of endogenous NO production in the leaves showed that although NaCl could trigger NO production in the three *B. napus* genotypes, the induction of NO synthesis was more pronounced in the leaves of the tolerant hybrid plants, compared with the two sensitive parent materials (Figure 4D). Also, a peaking point of NO production was observed after 12 h of stress in the three materials. The above results can explain partly why the hybrid of *B. napus* exhibited more tolerance than two parent materials when challenged with NaCl stress, since NO has been found to be closely positively associated with salinity tolerance in plants (Zhao et al., 2004, 2007).

In order to further elucidate the role of endogenous NO accumulation involved in heterosis under salt stress in *B. napus*, cPTIO (a NO scavenger) and SNP (a NO-releasing compound) were either used. Although cyanide in SNP has drastic effect on the mitochondria (Thomas et al., 2009), many reports revealed that negative effects of appropriate concentration of SNP is very little (Bai et al., 2011; Xie et al., 2014; Zhang et al., 2018). By using LSCM, subsequent results revealed that the addition of SNP could further intensify NO production in the presence or absence of NaCl stress, and these responses were more pronounced in the hybrid than in the two parent materials (Figure 4E), further implying that induced endogenous NO level is necessary for heterosis of salinity tolerance. Meanwhile, contrasting changes were observed in the presence of cPTIO. The above results matched with the changes in the contents of chlorophyll (Figure 5A) and proline (Figure 6A), reflecting that NR-dependent NO level was positively correlated with plant tolerance against NaCl stress. Overall, our pharmacological results suggested the important function of endogenous NO in the heterotic tolerance response to salt stress.

Unlike the signaling function of ROS in the early period of stressed conditions (Xie et al., 2011), overaccumulation of ROS during the late stage of abiotic stress causes oxidative stress in plants. Previous reports revealed that environmental stress-induced ROS generation could be modulated by NO, which could re-establish redox homeostasis and decrease oxidative damage (Turkan, 2018; Zhang et al., 2018). Therefore, the alleviated oxidative damage in hybrid plants might be caused by NO-decreased ROS levels (Figures 2A–D, 4).

Since NO could influence various metabolism pathways in plants, including photosynthesis upon abiotic stresses (Besson-Bard et al., 2008; Bai et al., 2011), representative metabolic processes associated with the NO control of salt stress tolerance in hybrid plants are compared and discussed below. It is well documented that salt stress can cause inhibition of chlorophyll biosynthesis (Duan et al., 2016; Chen et al., 2017). Previous reports also showed that hybrid plants displayed obviously photosynthetic potential in different plant species, such as *Arabidopsis* (Fujimoto et al., 2012) and *Physocarpus amurensis* (Xu et al., 2018).

As a multiple functional gaseous signaling molecule, NO exerts its biological function through different ways, including the control of second messengers, interaction with protein kinases, or regulation of gene expression (Astier and Lindermayr, 2012). Besides, NO-dependent post-translational modifications, such as *S*-nitrosylation, are other ways to interact with other specific proteins, including the proteins in photosynthesis (Galetskiy et al., 2011; Tanou et al., 2012).

In the present study, proteomic profiles revealed that the addition of SNP had more pronounced significant effects on NaCl-increased representative chlorophyll synthetic protein levels in the hybrid plants (Table 2 and Figure 5B), and these proteins include some responsible for the biosynthesis of tetrapyrrole (HEMC1, A0A078G803) and chlorophyll a/b (PPOX, A0A078FSF5; CHLM, A0A078HA16; PORB, A0A078GUT8; and PCB2, A0A078FHJ5) (Tanaka and Tanaka, 2006). Contrasting changes were observed when cPTIO was exogenously applied together. We also noticed that the amounts of these protein levels in F1 plants were higher than those in the parent materials, in the presence or absence of NaCl stress alone (Supplementary Table 2). Combined with similar changing tendencies in chlorophyll contents (Figure 5A) and corresponding mRNA abundance (Figure 5C), we further proposed that heterosis-improved chlorophyll synthesis upon salinity might require the participation of endogenous NO.

The accumulation of proline plays an important role in plant defense against abiotic stress (Ribarits et al., 2007; Szabados and Savouré, 2010; La et al., 2020). Previous results revealed that exogenous SNP could enhance proline production *via* modulating its synthesis and degradation genes (Su et al., 2018). As anticipated, compared with parental lines, the increased proline synthesis was further intensified in salt-stressed hybrid plants, confirmed by the increased protein levels and transcriptional abundance of P5CS1/2 and P5CR (Figures 6A,B, Table 3, and Supplementary Table 2). Consistently, the reduction in proline degradation in F1 plants, represented as the changes in PDH protein and transcriptional levels, was also observed. Subsequent results showed that proline metabolism was further stimulated by the administration with SNP, but suppressed by cPTIO, especially in hybrid plants (Table 3 and Figure 6). A similar change in proline production was observed in plant hybrid tolerance against salt stress (Pérez-Alfocea et al., 1994, 1996). Therefore, combined with the results shown in Figure 4, the increased proline accumulation in hybrid *B. napus* seedlings under salt stress was likely dependent on the stimulated NO production.

The TCA cycle is one of the key ways of cellular processes of carbon and nitrogen metabolism in plant mitochondria and is also important for fueling respiratory electron transport and, thus, ATP synthesis (Taylor et al., 2004; Kosová et al., 2011). Most importantly, the TCA cycle could provide the energy and carbon skeleton for the protein synthesis related to chlorophyll (Nabeta et al., 1998; Porra and Scheer, 2000) and proline biosynthesis (Hildebrandt et al., 2015). Previous biochemical and proteomic analysis revealed that TCA cycle enzymes were sensitive to abiotic and oxidative stresses (Hossain et al., 2012). In order to survive under salt stress conditions, a large amount of ATP is required to modulate a series of adaptive metabolic processes, including maintaining ion homeostasis and charge balance, *de novo* synthesis of stress-related and metabolism-related proteins, etc. (Sweetlove et al., 2002; Taylor et al., 2004; Duan et al., 2016). In this study, five proteins involved in the TCA cycle, namely PYD2 (A0A078FMN3), CS (A0A078IH04), ICDH (A0A078FVI0), OGD2 (A0A078FRZ0), and SCS (A0A078HSE9), were slightly suppressed after exposure to NaCl in hybrid plants (Table 4 and Supplementary Table 2). By contrast, these proteins were significantly downregulated in the two parental lines. Considering that NO can promote plant growth by enhancing the mitochondrial TCA cycle (Mao et al., 2018), SNP and cPTIO were used to probe the casual link between NO-mediated TCA cycle and heterosis under salt stress. The results showed that the application of exogenous SNP increased the expression of five TCA cycle relevant proteins and genes under salt stress in the hybrid plants (Table 4 and Figure 7), whereas the removal of endogenous NO with cPTIO brought about contrasting responses. Taken together, it was concluded that the TCA cycle might be the target of NO-mediated heterosis of salinity tolerance in *B. napus*, thus improving chlorophyll and proline synthesis upon NaCl stress (Figures 4A, 5A). Besides, we noticed that the data suggested that a significant fraction of the proteome is affected in this study (Figures 3, 5–7). In fact, proteins in cells could not perform their functions as single entities, but can work together in the context of networks (Dai et al., 2017).

## Conclusion

**FIGURE 8:**
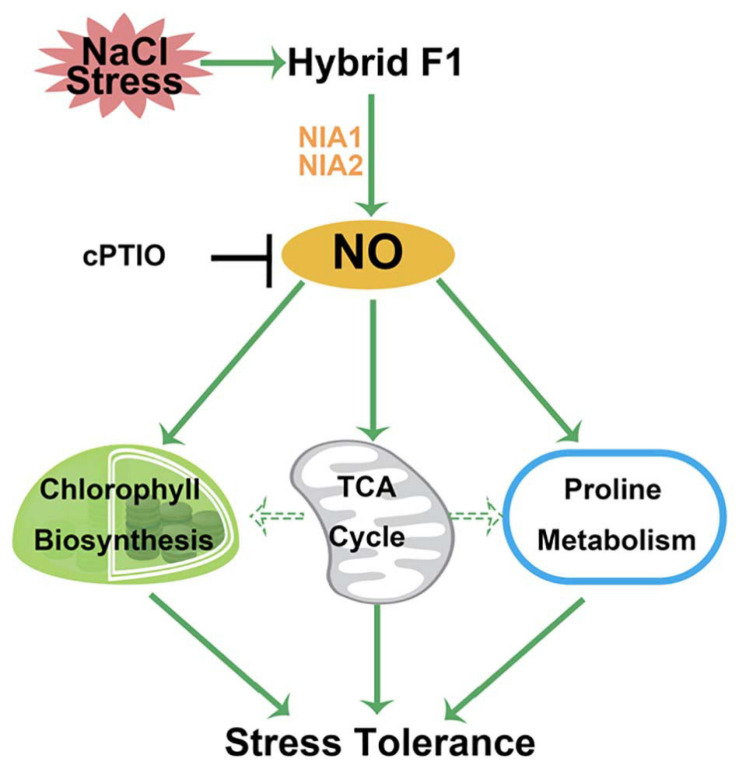
Schematic diagram of the potential role of NO in the heterosis of *B. napus* against salt stress at the proteomic and physiological levels. A rapid response of NO production catalyzed by NIA1/2 in hybrid is induced in response to salt stress. NO might control TCA cycle metabolism pathway, thus providing the energy and carbon skeleton for the protein synthesis related to chlorophyll and proline biosynthesis. Finally, salinity toxicity was alleviated. T bars, inhibition.

In conclusion, this study investigated the responses of a *B. napus* hybrid, produced by a cross between NJ4375 and MB1942, upon NaCl stress. Using proteomic and pharmacological approaches, we discovered the important roles of endogenous NO in the heterosis of salinity tolerance, which was dependent on the activation of chlorophyll and proline metabolism as well as TCA cycle. Based on these results, a working model was summarized to explain the detailed mechanism of NO-dependent heterotic tolerance upon salt stress (Figure 8). Certainly, the casual link between NO and ROS in heterosis is another future work. Our results not only suggested that the mechanism by NO-dependent heterotic tolerance to salt tress is achieved, but also further implied the central roles of endogenous NO functioning in both developmental processes and stress responses in crops.

## Data Availability Statement

The datasets presented in this study can be found in online repositories. The names of the repository/repositories and accession number(s) can be found below: Proteomics Identifications Database (PRIDE), accession no: PXD023601.

## Author Contributions

YZ, CD, and WS designed the experiment. YZ, PC, JW, YL, MW, CD, SW, RG, and HP performed the research. YZ, PC, CD, and WS analyzed the data. YZ, DA, and WS wrote and revised the manuscript, with contribution and approval from all authors.

## Conflict of Interest

The authors declare that the research was conducted in the absence of any commercial or financial relationships that could be construed as a potential conflict of interest.

## Footnotes

1 http://www.uniprot.org

2 http://www.omicsbean.cn

3 http://bioinformatics.psb.ugent.be/webtools/Venn/

4 https://www.genoscope.cns.fr/brassicanapus

## Supplementary Material

Supplementary Figure 1 The NO levels at germination stage. **(A)** After seeds germinated for 3 days, the roots were loaded with 4-amino-5-methylamino-2′,7′-difluorofluorescein diacetate (DAF-FM DA), and detected by laser scanning confocal microscopy (LSCM). DAF-FM DA-dependent fluorescence densities according to panel **(A)** were given panel **(B)**. Values are the means ± SD for three independent experiments. One-way ANOVA analyses were conducted according to Duncan’s multiple range test. Different letters denote significant differences at *P* < 0.05.

Supplementary Table 1 The sequences of primers for qPCR.

Supplementary Table 2 Total number of identified proteins.
