# Supplementary material for: Nitric Oxide Is Associated With Heterosis of Salinity Tolerance in Brassica napus L
Source: Front Plant Sci. 2021 May 28;12:649888. doi: 10.3389/fpls.2021.649888 (PMC8194068; doi:10.3389/fpls.2021.649888)
Supplement: Supplementary Figure 1 — The NO levels at germination stage. (A) After seeds germinated for 3 days, the roots were loaded with 4-amino-5-methylamino-2′,7′-difluorofluorescein diacetate (DAF-FM DA), and detected by laser scanning confocal microscopy (LSCM). DAF-FM DA-dependent fluorescence densities according to panel (A) were given panel (B). Values are the means ± SD for three independent experiments. One-way ANOVA analyses were conducted according to Duncan’s multiple range test. Different letters denote significant differences at P < 0.05. [file Data_Sheet_2.doc]

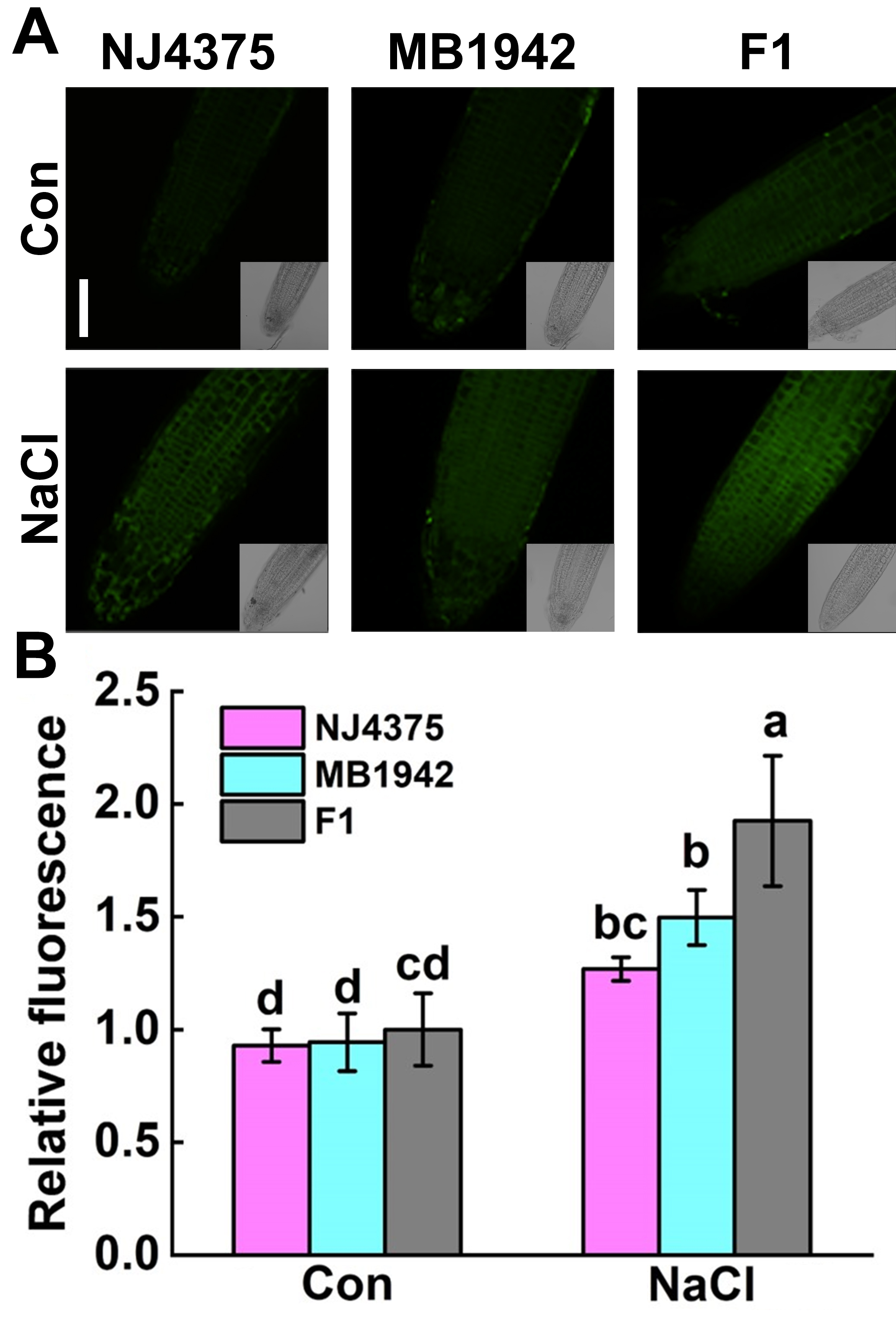
**Supplementary Figure 1** The NO levels at germination stage. **(A)** After seeds germinated for 3 d, the roots were loaded with 4-amino-5-methylamino-2’,7’-difluorofluorescein diacetate (DAF-FM DA), and detected by laser scanning confocal microscopy (LSCM). DAF-FM DA-dependent fluorescence densities according to **(A)** were given **(B)**. Values are the means ± SD for three independent experiments. One-way ANOVA analyses were conducted according to Duncan’s multiple range test. Different letters denote significant differences at *P* < 0.05.
